# Acinetobacter baumannii Nosocomial Meningitis Secondary to Neurosurgical Intervention: A Case Report

**DOI:** 10.7759/cureus.115707

**Published:** 2026-09-03

**Authors:** Inssaf Haddi, Yousra Boughalem, Youssef El Kamouni, Lamiae Arsalane, Said Zouhair

**Affiliations:** 1 Department of Microbiology, Avicenne Military Hospital, Marrakech, MAR

**Keywords:** cerebrospinal fluid (csf), craniopharyngioma, multidrug-resistant acinetobacter, nosocomial bacterial meningitis, acinetobacter baumannii

## Abstract

Nosocomial meningitis is a serious complication of neurosurgical procedures, associated with high morbidity and mortality, as well as significant diagnostic and therapeutic challenges. Among the causative pathogens, *Acinetobacter baumannii* *(A. baumannii)* is particularly concerning due to its ability to survive for prolonged periods in the hospital environment, its opportunistic virulence, and, especially, its tendency to develop multidrug resistance to antimicrobial agents. Diagnosis is often difficult in the postoperative setting, particularly when the cerebrospinal fluid (CSF) is altered as a result of the surgical procedure. Rapid identification of the causative agent and early initiation of appropriate antibiotic therapy are essential to limit complications and improve patient prognosis.

We report the case of a 27-year-old patient with a history of surgically treated craniopharyngioma complicated by anterior hypopituitarism. Three weeks after surgery, he was admitted to the ICU with suspected postoperative meningitis due to the onset of behavioral disturbances associated with nasal discharge, without a fever. Brain CT was normal. CSF analysis, obtained by lumbar puncture under strict aseptic conditions, revealed a turbid fluid with leukocytosis of 5,760 cells/mm³, predominantly composed of neutrophils, along with elevated protein levels (5.7 g/L) and low glucose levels (0.05 g/L). Direct examination after Gram staining showed Gram-negative coccobacilli with a marked inflammatory reaction. CSF culture grew a multidrug-resistant (MDR) strain of *A. baumannii*, which was sensitive only to colistin.

The patient received antibiotic therapy with colistin, administered intravenously and intrathecally for 18 days, with initial neurological improvement and sterilization of the CSF by the 15th day of treatment. The subsequent course was unfavorable, marked by the development of acute respiratory distress syndrome (ARDS) secondary to ventilator-associated pneumonia caused by methicillin-resistant *Staphylococcus aureus* (MRSA), complicated by refractory septic shock, which ultimately led to his death after one month of hospitalization in the ICU. This report highlights the severity of nosocomial meningitis caused by MDR *A. baumannii* and underscores the importance of implementing infection prevention measures in healthcare settings.

## Introduction

Nosocomial meningitis represents a formidable complication of neurosurgical procedures, particularly when it occurs in the postoperative setting. Among the pathogens involved, *Acinetobacter baumannii (A. baumannii)* is of particular concern due to its ability to survive in the hospital environment, its opportunistic virulence, and, above all, its marked tendency to develop multiple antibiotic resistances. In particular, *A. baumannii* may exhibit multidrug-resistant (MDR) or extensively drug-resistant (XDR) profiles, including resistance to β-lactams and carbapenems, which can considerably limit therapeutic options. Nosocomial meningitis caused by *A. baumannii* poses a significant diagnostic and therapeutic challenge and is associated with high morbidity and mortality, as diagnosis is often difficult and treatment remains controversial [1].

Diagnosis remains complex in the presence of cerebrospinal fluid (CSF) that has been altered by the surgical procedure, making interpretation difficult. According to the available literature, rapid diagnosis and appropriate antibiotic therapy are essential, as they help limit morbidity and mortality [2]. However, the occurrence of this condition in the postoperative neurosurgical setting is relatively rare, and the frequent multidrug resistance of *A. baumannii* strains represents a major therapeutic challenge, given the limited number of molecules that manage to diffuse into the meninges [3]. Clinical signs suggestive of this condition include fever and deterioration of consciousness, as well as headache, seizures, and signs of meningeal irritation. However, these symptoms are often nonspecific and may not be present in the postoperative setting [4].

We report a case of post-surgical nosocomial *A. baumannii* meningitis due to a craniopharyngioma with antehypophyseal insufficiency. This report highlights the diagnostic difficulties associated with postoperative meningitis and the therapeutic challenges posed by MDR *A. baumannii*, emphasizing the importance of early recognition and appropriate antimicrobial management.

## Case presentation

The patient was a 27-year-old male with a history of surgically treated craniopharyngioma and antehypophyseal insufficiency. Three weeks later, he was admitted to the ICU with suspected postoperative meningitis due to behavioral disturbances, an absence of fever, and nasal discharge. He underwent a cerebral CT scan, as shown in Figure 1, at the time of admission to the ICU. It showed no significant intracranial abnormality. This imaging was performed to assess for intracranial abnormalities in the context of his neurological deterioration.

**Figure 1:**
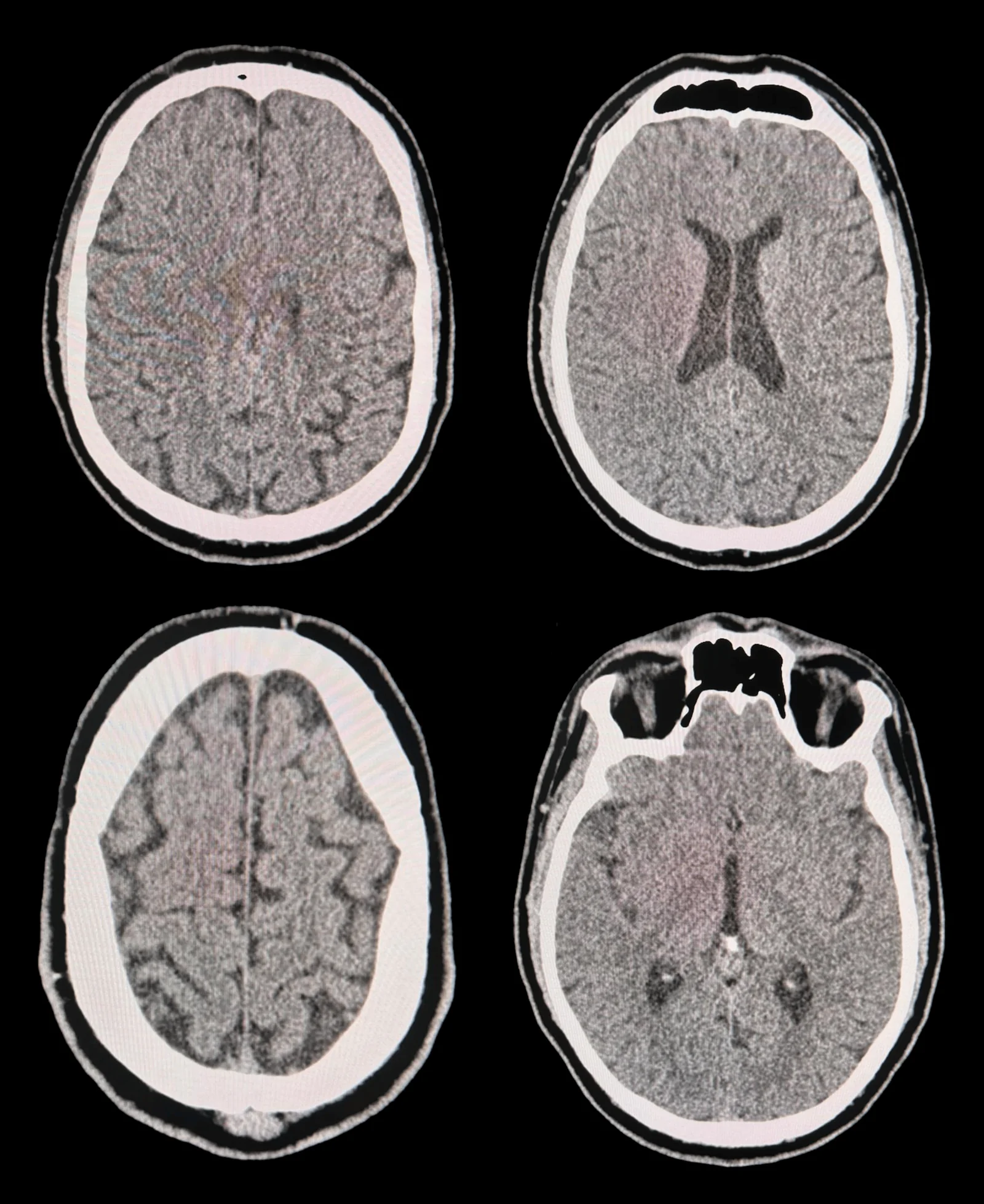
Cerebral CT scans performed at admission to the ICU, showing no significant intracranial abnormality The images are presented to illustrate the initial neuroimaging findings during the postoperative episode CT: computed tomography; ICU: intensive care unit

A full biological workup of CSF obtained by lumbar puncture, performed under rigorous aseptic operative conditions, revealed turbid fluid containing 5,760 leukocytes/mm³, predominantly neutrophils. Hyperproteinorachia (5.7 g/L) and hypoglycorachia (0.05 g/L) were found; concomitant blood glucose was 1.16 g/L, with a C-reactive protein (CRP) level of 227.8 mg/L.

Direct examination of the CSF after Gram staining revealed the presence of bacteria in the form of Gram-negative coccobacilli, accompanied by an intense inflammatory reaction characterized by an abundance of neutrophilic polymorphonuclears (PNNs) (Figure 2).

**Figure 2:**
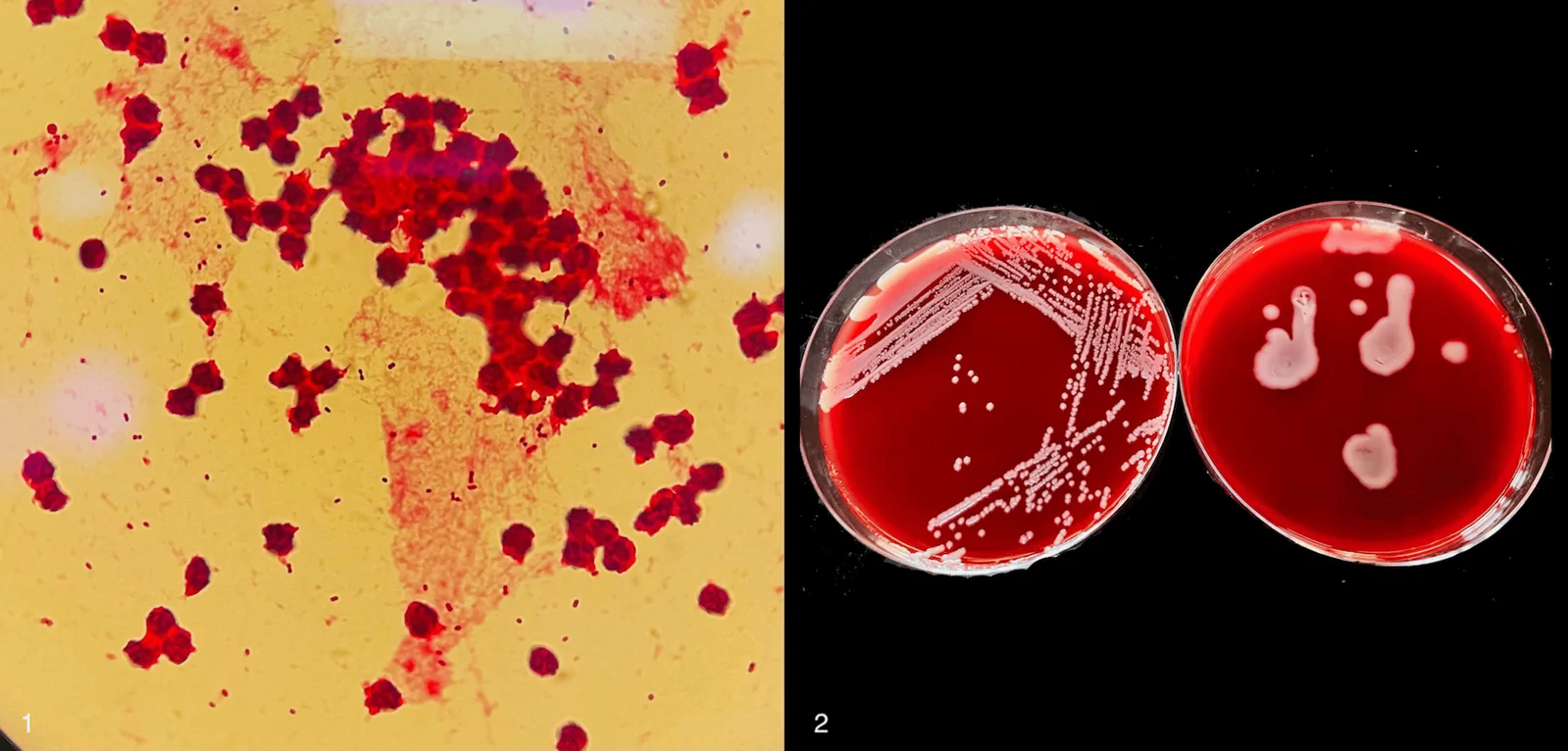
Direct examination and culture of the CSF (1) Direct examination of the CSF shows an inflammatory reaction with coccobacilli. (2) Culture of the CSF on blood agar, inoculated at three points and by streaking, is positive after incubation for 24 hours at 37 °C under aerobic conditions, confirming the growth of *Acinetobacter baumannii*​​​​​​. The CSF sample was obtained by lumbar puncture during the episode of postoperative meningitis CSF: cerebrospinal fluid

The CSF culture isolated a strain of MDR *A. baumannii *sensitive only to colistin. The patient underwent antibiotic therapy with intravenous and intrathecal colistin (9,000,000 IU daily for 18 days), with initial neurological improvement and CSF sterilization 15 days after the initiation of antibiotic treatment. He was also treated with a therapeutic dose of low-molecular-weight heparin (LMWH) (40 mg daily), followed by direct oral anticoagulants (DOACs) (20 mg daily), and levothyroxine and hydrocortisone to compensate for his antehypophyseal insufficiency.

The course was marked by the onset of respiratory arrest, for which the patient was intubated, mechanically ventilated, and sedated. The patient subsequently developed acute respiratory distress syndrome (ARDS) secondary to methicillin-resistant *Staphylococcus aureus* (MRSA) pneumonia while mechanically ventilated. The course was unfavorable, with the onset of septic shock refractory to high-dose noradrenaline, and the patient died after a one-month stay in the ICU.

## Discussion

*A. baumannii* is a Gram-negative, polymorphic, non-fermentative, non-spore-forming coccobacillus that may be encapsulated in certain pathological specimens. It is strictly aerobic, catalase-positive, and oxidase-negative [3]. It is part of the commensal flora of the skin, particularly in humid areas, and the digestive tract. Despite its limited intrinsic virulence, it has been implicated in serious nosocomial infections, particularly in ICUs and immunocompromised patients [5]. Its prolonged ability to survive on surfaces favors skin colonization in intensive care patients, while its transmission relies mainly on hand carriage, particularly in epidemic situations [6].

In clinical practice, *Acinetobacter* infections are frequently associated with surgical procedures or the use of artificial medical devices, with infection generally occurring after an initial colonization phase. This process is facilitated by various risk factors, particularly in the ICU, where repeated postoperative manipulations, combined with the use of endotracheal tubes and intravascular, ventricular, or urinary catheters, facilitate colonization by opportunistic bacteria such as *Acinetobacter* [6]. Our patient developed nosocomial meningitis with *A. baumannii* 21 days after surgery. Many risk factors may explain the occurrence of this severe infection: the neurosurgical procedure performed for a craniopharyngioma, anterior pituitary insufficiency potentially predisposing the patient to reduced resistance to infection, the suspicion of CSF leak suggested by nasal discharge, and the prolonged duration of the surgical procedure. The prolonged stay in intensive care and repeated manipulation of the patient also favored colonization, followed by infection, by MDR bacteria. The isolation of an MDR strain of *A. baumannii*, sensitive only to colistin, supports the nosocomial nature and severity of the infection, which complicated treatment [7].

Nosocomial meningitis caused by *A. baumannii* remains relatively rare [8] and is only exceptionally reported following neurosurgical interventions. In a review of the literature, Krol et al. reported 30 well-documented cases, totaling 120 patients, recorded between 1962 and 2007 [7]. Other authors have also reported limited series, including series ranging from one to 25 cases of nosocomial *Acinetobacter* meningitis following neurosurgical procedures [9-10]. *A. baumannii *strains are characterized by their high resistance to most antibiotics [3]. In our case, the isolate was sensitive only to colistin, a molecule that has been successfully used intravenously or intrathecally to treat MDR *A. baumannii *infections [11-12].

In this case, the presence of *A. baumannii* was confirmed by its isolation in pure culture from a CSF sample obtained by lumbar puncture, performed under strict aseptic conditions and in accordance with the recommendations of the Good Laboratory Practice Guide. Cytological analysis of the CSF revealed hypercellularity with a predominance of neutrophil cells (Figure 3), while biochemical examination showed hypoglycorrhachia associated with hyperproteinorrhachia (Table 1).

**Figure 3:**
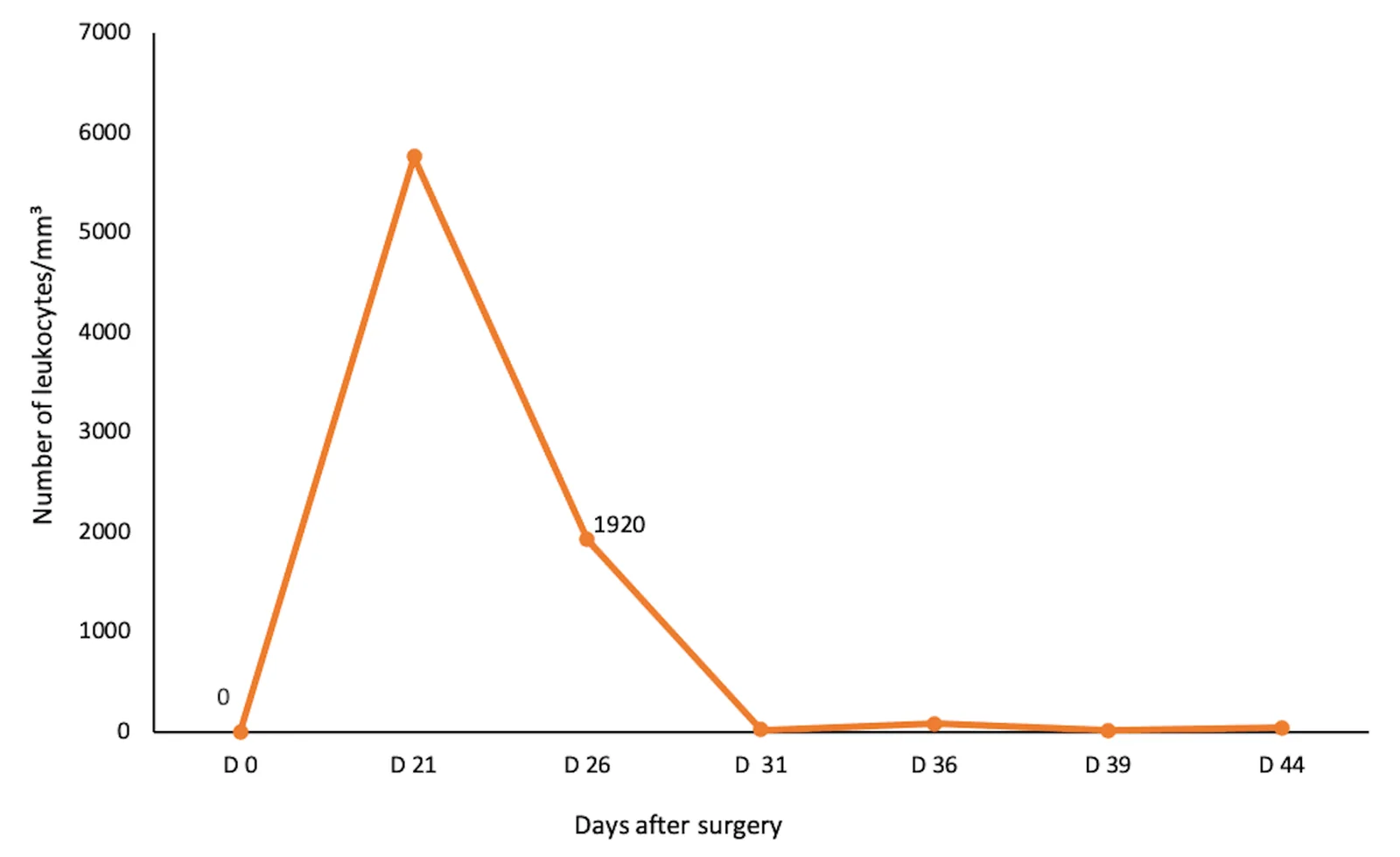
Evolution of CSF cytology during the postoperative meningitis episode The graph illustrates the changes in CSF leukocyte count over time, with a marked increase during the acute phase of infection followed by a progressive decrease during treatment CSF: cerebrospinal fluid

**Table 1:** CSF biochemical and macroscopic characteristics during the postoperative meningitis episode The CSF findings were consistent with bacterial meningitis, with marked hypoglycorrhachia and hyperproteinorrachia during the acute phase, followed by progressive normalization during treatment. The initial CSF sample (Day 1) was collected at the time of diagnosis of meningitis, 21 days after surgery CSF: cerebrospinal fluid

	Days after diagnosis of meningitis							
Variable	Day 1	Day 5	Day 10	Day 15	Day 18	Day 23	Day 30	Reference range
CSF appearance	Troubled rice water	Purulent	Clear	Troubled rice water	Clear rock water	Clear rock water	Clear rock water	Clear
Glycorrhachia (g/L)	0.05	< 0.05	0.82	0.32	0.28	0.34	0.43	0.5-0.8
Chlorurorachia (mmol/L)	115	132	137	123	129	125	116	120-130
Proteinorachy (g/L)	5.7	5.6	0.61	2.4	1.05	0.49	0.5	0.15-0.45

However, the clinical course was complicated, 10 days after the onset of meningitis, by the onset of documented nosocomial MRSA pneumonia. This complication, which is frequent in the ICU, can be attributed to the patient's persistent vulnerability, prolonged intubation, and invasive care, illustrating the high risk of multiple nosocomial infections in neurosurgical patients managed in intensive care [13]. The mortality rate associated with nosocomial *A. baumannii* infections in neurosurgery remains high. Some studies have reported mortality rates of 27% in the USA [7], 40% in Slovenia [9], and 15% in Australia [14]. In India, Gulati et al. observed a significantly higher mortality rate in patients with nosocomial meningitis than in those with MDR *A. baumannii* bacteremia [15].

In neurosurgery, the prevention of nosocomial infections, particularly those linked to patient handling and MDR bacteria, relies on the reinforcement of preventive measures. These include rigorous hand hygiene using hydro-alcoholic friction, combined with ongoing staff training, targeted isolation measures, and an effective surveillance program [16]. The combination of these interventions, coupled with the rational use of antibiotics, is a key element in reducing selection pressure and limiting the dissemination of resistant strains.

This report highlights the importance of maintaining a high level of clinical suspicion for postoperative meningitis, even in the absence of typical clinical signs such as fever. In the setting of neurosurgical patients, early CSF analysis and microbiological identification remain essential for guiding appropriate antimicrobial therapy, particularly when MDR organisms are suspected. The isolation of a strain susceptible only to colistin also illustrates the limited therapeutic options available in such infections and the need for individualized treatment based on antimicrobial susceptibility and central nervous system penetration.

## Conclusions

This case report illustrates the complexity of managing postoperative nosocomial meningitis, particularly given the poor diffusion of antibiotics in the CSF. In our case, despite treatment with intravenous and intrathecal colistin and CSF sterilization, the patient subsequently developed severe nosocomial MRSA pneumonia complicated by refractory septic shock and died. The prognosis remains generally unfavorable, with a high mortality rate. The report highlights the importance of early diagnosis, appropriate antimicrobial therapy, antimicrobial stewardship, and rigorous infection-prevention measures in patients at risk of MDR infections. Close collaboration between clinicians, radiologists, and microbiologists is essential to optimize treatment strategies and improve patient outcomes.
